# The MAX IV storage ring project

**DOI:** 10.1107/S1600577514011503

**Published:** 2014-08-27

**Authors:** Pedro F. Tavares, Simon C. Leemann, Magnus Sjöström, Åke Andersson

**Affiliations:** a MAX IV Laboratory, Lund University, PO Box 118, SE-221 00 Lund, Sweden

**Keywords:** storage ring, synchrotron light source, multibend achromat

## Abstract

The MAX IV facility, currently under construction in Lund, Sweden, features two electron storage rings operated at 3 GeV and 1.5 GeV and optimized for the hard X-ray and soft X-ray/VUV spectral ranges, respectively. A 3 GeV linear accelerator serves as a full-energy injector into both rings as well as a driver for a short-pulse facility, in which undulators produce X-ray pulses as short as 100 fs. The 3 GeV ring employs a multibend achromat (MBA) lattice to achieve, in a relatively short circumference of 528 m, a bare lattice emittance of 0.33 nm rad, which reduces to 0.2 nm rad as insertion devices are added. The engineering implementation of the MBA lattice raises several technological problems. The large number of strong magnets per achromat calls for a compact design featuring small-gap combined-function magnets grouped into cells and sharing a common iron yoke. The small apertures lead to a low-conductance vacuum chamber design that relies on the chamber itself as a distributed copper absorber for the heat deposited by synchrotron radiation, while non-evaporable getter (NEG) coating provides for reduced photodesorption yields and distributed pumping. Finally, a low main frequency (100 MHz) is chosen for the RF system yielding long bunches, which are further elongated by passively operated third-harmonic Landau cavities, thus alleviating collective effects, both coherent (*e.g.* resistive wall instabilities) and incoherent (intrabeam scattering). In this paper, we focus on the MAX IV 3 GeV ring and present the lattice design as well as the engineering solutions to the challenges inherent to such a design. As the first realisation of a light source based on the MBA concept, the MAX IV 3 GeV ring offers an opportunity for validation of concepts that are likely to be essential ingredients of future diffraction-limited light sources.

## Abstract 2

The design of the MAX IV 3 GeV ultralow-emittance storage ring is presented and the implementation of solutions to the technological challenges imposed by the compact multi-bend achromat lattice are described.

## 1. Introduction   

The MAX IV facility, currently under construction in Lund, Sweden, is the first of a new generation of storage-ring-based synchrotron light sources which employ a multibend achromat lattice to reach emittances in the few hundred pm rad range in a circumference of a few hundred metres, thus enabling the realisation of a new class of experiments which are critically dependent on source brightness and transverse coherence.

Central to the MAX IV design concept is the notion that the diverse needs of the user community are difficult to satisfy with a single source without compromising performance. In fact, the scientific case for the MAX IV project (MAX IV, 2006 ▶) requires high average brightness over a wide spectral range from infrared to hard X-rays as well as intense short X-ray pulses in the fs range. An analysis (MAX IV, 2006 ▶) of alternative solutions to meet those requirements led to the conclusion that storage-ring-based sources are likely to continue to be the workhorse of synchrotron-radiation-based research for the foreseeable future and that recent advances in accelerator lattice design and engineering development in key subsystems indicated the possibility of a substantial decrease in storage ring emittance, bringing those sources closer to the diffraction limit at X-ray wavelengths. Moreover, the growing demand for temporally as well as spatially coherent radiation pointed to the fact that free-electron lasers will also open new research opportunities.

**Figure 1:**
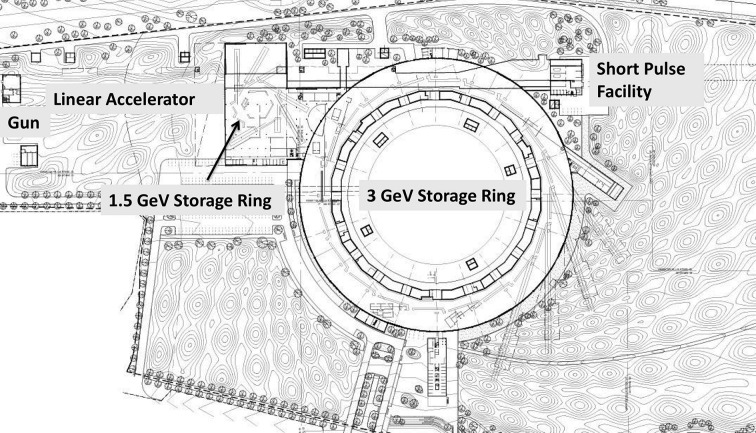
Overview of the MAX IV facility.

All of those considerations were included in a facility-wide optimization procedure that led to a design (MAX IV, 2010 ▶) based on three sources sharing a common site and infrastructure (Fig. 1 ▶):

(i) Two electron storage rings operating at different energies (1.5 GeV and 3 GeV) in order to cover a wide photon energy range in an optimized way with short-period insertion devices.

(ii) A linear accelerator which acts as a full-energy injector into both rings and provides electron pulses with duration below 100 fs to produce X-rays by spontaneous emission in the undulators of a short-pulse facility (SPF) (Werin *et al.*, 2009 ▶; Thorin *et al.*, 2011 ▶). The 3 GeV linear accelerator also allows a future upgrade to a fully coherent free-electron laser source based on seeding and/or cascading (Čutić *et al.*, 2010 ▶; Curbis *et al.*, 2013 ▶).

The 3 GeV ring (Leemann *et al.*, 2009 ▶; Eriksson *et al.*, 2011 ▶) is optimized for the production of high-brightness hard X-ray beams and features a 20-fold seven-bend achromat lattice, reaching a bare lattice emittance of 0.33 nm rad, which is further reduced to 0.2 nm rad when insertion devices are added. In order to reach such a low emittance in a circumference of only 528 m, a compact magnet design is mandatory. This implies the use of small magnet gaps (Johansson *et al.*, 2011 ▶), which allows reaching larger integrated gradients in shorter magnets and reduces the minimum required distance between consecutive magnets. Moreover, these compact magnets are built as integrated units in which the bending magnet poles and quadrupole pole roots are machined out of a pair of iron blocks, which are assembled together, each unit holding all the magnets of a complete cell. This concept leads to alignment accuracy within a cell being determined by machining and assembly accuracy, rather than fiducialization methods and also makes for high natural vibration frequencies of the units, thus reducing the sensitivity of the magnets to the environmental vibrational noise. Finally, the integrated magnet concept allows for streamlined installation and system testing.

The compact magnet design leads to narrow low-conductance vacuum chambers (Al-Dmour *et al.*, 2011 ▶), which necessitate distributed pumping and distributed absorption of the heat load from synchrotron radiation. The heat load problem is dealt with by choosing copper as the chamber material and providing water cooling along the extended region over which the synchrotron radiation heat is deposited, whereas distributed pumping is provided by non-evaporable getter (NEG) coating of the chamber’s inner surface. As a result, the number of required lumped pumps and absorbers is significantly reduced with a corresponding reduction in cost and complexity.

The reduced chamber dimensions lead to an increased risk of collective instabilities (Tavares *et al.*, 2011 ▶), such as coupled bunch instabilities driven by the resistive wall impedance. A key ingredient in facing that problem is the use of passively operated harmonic cavities, which lengthen the bunches, reduce the electron density, help keep the heat load from beam-induced fields on vacuum components down to acceptable levels, and increase the incoherent synchrotron frequency spread that enhances Landau damping of coherent instabilities.

The RF system (Andersson *et al.*, 2011 ▶) operates at 100 MHz and uses capacitive-loaded normal conducting cavities, of the same type as previously developed for MAX II and MAX III. The choice of RF frequency allows a large bucket height with relatively low RF voltage and power to be achieved, which can be obtained from standard high-efficiency RF transmitters largely used in telecommunications, leading to low investment and operation costs. Moreover, the cavity design pushes the frequencies of the first higher-order modes (HOMs) of the cavity to about four times the fundamental mode frequency, so as to reduce the overlap of the cavity impedance spectrum with the spectrum of the lengthened bunches.

The 1.5 GeV ring (MAX IV, 2010 ▶; Leemann, 2012*c* ▶) will replace the existing MAX II (Andersson *et al.*, 1994 ▶) and MAX III (Sjöström *et al.*, 2009 ▶) rings in delivering UV, soft X-ray and infrared radiation. With about the same circumference (96 m) as MAX II, the 1.5 GeV ring will deliver a smaller emittance (6 nm rad) than its predecessor by applying the same compact multipurpose magnet design concept (Johansson, 2011 ▶) as in the 3 GeV ring to a 12-fold DBA lattice. Here, two gradient dipole magnets, three combined quadrupole/sextupole magnets as well as four pure sextupoles and four combined trim sextupoles/orbit correctors are all integrated into a single iron block pair comprising a full DBA arc. An exact copy of the 1.5 GeV ring is being built at the Polish laboratory Solaris (Bocchetta *et al.*, 2012 ▶).

## 2. Lattice and optics   

The 3 GeV storage ring will serve as the main radiation source of the MAX IV facility. In order to generate high-brightness hard X-rays with state-of-the-art insertion devices (IDs), an ultralow-emittance design was targeted. One simple and robust method to achieve ultralow emittance is the use of a multibend achromat (MBA) lattice (Einfeld & Plesko, 1993 ▶; Joho *et al.*, 1994 ▶; Einfeld *et al.*, 1995 ▶; Kaltchev *et al.*, 1995 ▶). The MBA exploits the inverse cubic dependence of emittance on the number of bending magnets.

By choosing a very small bending angle per dipole the emittance can be dramatically reduced. By introducing a vertically focusing gradient in the dipoles the emittance is further reduced (the emittance scales inversely with the horizontal damping partition 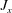) while the dispersion is limited to small values without requiring any extra space for vertically focusing quadrupoles. Because of the resulting low dispersion, the MBA lattice allows the use of narrow vacuum chambers without limiting momentum acceptance. This in turn enables narrow magnet gaps and hence magnets with strong gradients can become very compact (additionally, the compact magnets reduce the power consumption and hence the running cost). The compact magnets allow for a shorter unit cell; thus the number of unit cells for a given circumference can be increased. This in turn allows to further reduce the bend angle per unit cell which leads to even lower emittance (and in addition reduces the radiation heat load on the vacuum chamber). Thus, the MBA design approach closes a positive feedback cycle.

Before the MBA concept was first applied to a light source at MAX IV, this concept had been suggested for booster synchrotrons (Mülhaupt, 1994 ▶) of which several operate successfully today (Joho *et al.*, 2006 ▶; Georgsson *et al.*, 2004 ▶; Benedetti *et al.*, 2008 ▶). Distributing many sextupoles and/or making use of combined-function magnets throughout the MBA lattice allows the chromaticity to be corrected where it is generated (Klotz & Mülhaupt, 1992 ▶) giving large dynamic aperture and good off-energy performance. By introducing octupoles alongside the many sextupoles and carefully balancing non-linear magnet families, the non-linear optics can be tuned for large momentum acceptance (MA) and dynamic aperture (DA) providing both long Touschek lifetime and high injection efficiency despite the very low emittance (Leemann *et al.*, 2009 ▶; Leemann & Streun, 2011 ▶).

From its initial proposal in 2002 (Eriksson, 2002 ▶), the MAX IV 3 GeV storage ring lattice went through several iterations (Tarawneh *et al.*, 2003 ▶; Eriksson *et al.*, 2007 ▶, 2008 ▶) until a finalized version (Leemann *et al.*, 2009 ▶; MAX IV, 2010 ▶) was funded in 2010. The optics were subsequently refined (Leemann, 2011*a* ▶,*b* ▶) and a few minor modifications were made as a result of detailed magnet and vacuum systems engineering (Leemann, 2011*c* ▶, 2012*d* ▶). Further optics optimization is ongoing both in terms of choice of operational parameters (Leemann & Eriksson, 2013 ▶) as well as further modifications to user optics (Leemann & Eriksson, 2014 ▶).

### 2.1. Linear optics   

**Figure 2:**
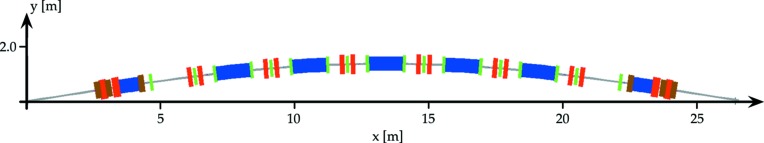
Schematic of one of the 20 achromats of the MAX IV 3 GeV storage ring. Magnets indicated are gradient dipoles (blue), focusing quadrupoles (red), sextupoles (green) and octupoles (brown).

The MAX IV 3 GeV storage ring consists of 20 seven-bend achromats separated by 4.6 m long straight sections for IDs. An overview of one MAX IV achromat is shown in Fig. 2 ▶. Each of the achromats consists of five unit cells and two matching cells. The unit cells have a 3° bending magnet, while the matching cells at the ends of the achromat have a 1.5° soft-end bending magnet. In these soft-end dipoles, the magnetic field drop-off towards the long straight reduces the amount of radiation hitting a downstream ID therefore facilitating the design of superconducting IDs.^1^ All dipoles contain a vertically focusing gradient. The matching cells contain dedicated quadrupole doublets in order to match the achromat optics to the ID in the long straight. Each achromat also contains two 1.3 m short straights that separate the matching cells from the unit cells. The short straights are used for RF cavities and diagnostics so that all long straights but the injection straight are available for installation of IDs.

**Figure 3:**
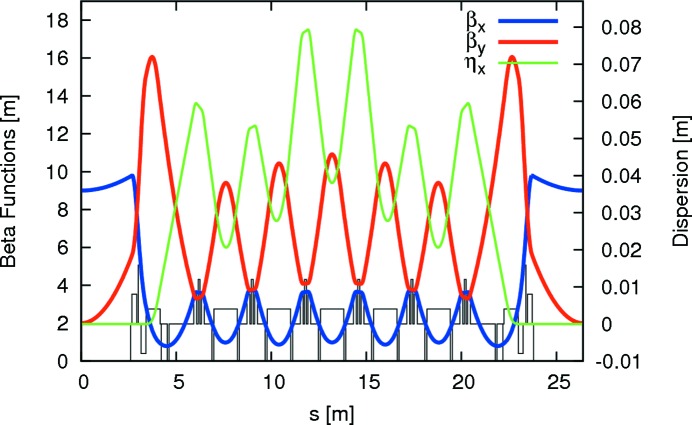
β functions and dispersion for one achromat of the MAX IV 3 GeV storage ring. Magnet positions are indicated at the bottom.

Since the vertical focusing is performed by the gradient dipoles, dedicated quadrupoles are, apart from ID matching (*cf.* §2.3), only required for horizontal focusing. Horizontally focusing quadrupoles are installed between the cells of the achromat in pairs of two where the two quadrupoles are installed on either side of a sextupole magnet. There are two families of focusing quadrupoles, one in the unit cells and one in the matching cells. Adjustment of the vertical focusing is performed by exciting a current in the pole-face strips (PFSs) that are installed in all dipoles. Such a lattice leads to very compact optics with strong focusing, low β functions, and very small peak dispersion. The optics for one achromat are displayed in Fig. 3 ▶ and ring parameters are given in Table 1 ▶.

The working point was chosen away from systematic resonances so that both fractional tunes are just above the integer and away from the most dangerous resonances. With the working point held constant during operation (*cf.* §2.3), the non-linear optics can be adjusted to minimize the chromatic and amplitude-dependent tune shifts (ADTSs), therefore keeping the tunes of most stored beam particles clear of dangerous resonances. This shall be explained in the next section.

### 2.2. Non-linear optics   

Despite comparably relaxed linear optics, the non-linear optics of such a MBA lattice are demanding. The strong focusing gives rise to large negative natural chromaticities that need to be corrected to prevent head–tail instability. This can be performed with chromatic sextupoles. Because of the low dispersion in the MBA these sextupoles tend to become very strong. Although this is not a concern for the magnet design (the 25 mm nominal magnet bore allows strong gradients), it presents an optics design challenge as such strong sextupoles give rise to pronounced non-linear amplitude-dependent behaviour, which can limit both DA and MA. The most common approach is to install several additional families of sextupoles separated by appropriate phase advances in an attempt to cancel resonance driving terms and limit chromatic tune shifts (Bengtsson, 1988 ▶, 1997*a* ▶; Streun, 2012 ▶).

The MAX IV 3 GeV storage ring contains five sextupole families, three focusing and two defocusing. The focusing sextupoles are installed between the focusing quadrupoles in the unit cells. This puts these sextupoles at locations with comparably large horizontal β function and dispersion. The defocusing sextupoles are installed as close as possible to the maximum of the product of dispersion and vertical β: unit cell dipoles are flanked on either side by a defocusing sextupole of one family while the defocusing sextupoles in the matching cells are installed in the short straights right next to the matching cell soft-end dipole. In this way, sextupoles compensate chromaticity where it is created thus limiting chromatic β beating (Mülhaupt, 1994 ▶). Because of the large number of installed sextupoles and the small magnet gap, the sextupoles can be kept short.

Sextupole optimization was performed with the codes *OPA* (Streun, 2010 ▶) and *Tracy-3* (Bengtsson, 1997*b* ▶). The linear chromaticities were corrected to +1.0 in both planes^2^ and the first-order resonance driving terms along with second- and third-order chromaticity were minimized as detailed by Streun (2012 ▶). However, amplitude-dependent tune shifts are only corrected as a second-order effect in sextupoles, therefore requiring a lot of sextupole gradient strength and in turn driving resonances and chromatic tune shifts. This can necessitate extra sextupoles and/or increased sextupole gradients in order to keep first-order terms in check. Apart from leading to a potential run-away problem, this is a delicate balance that is easily disturbed by IDs, alignment errors and higher-order multipoles, all of which exist in a real machine.

**Figure 4:**
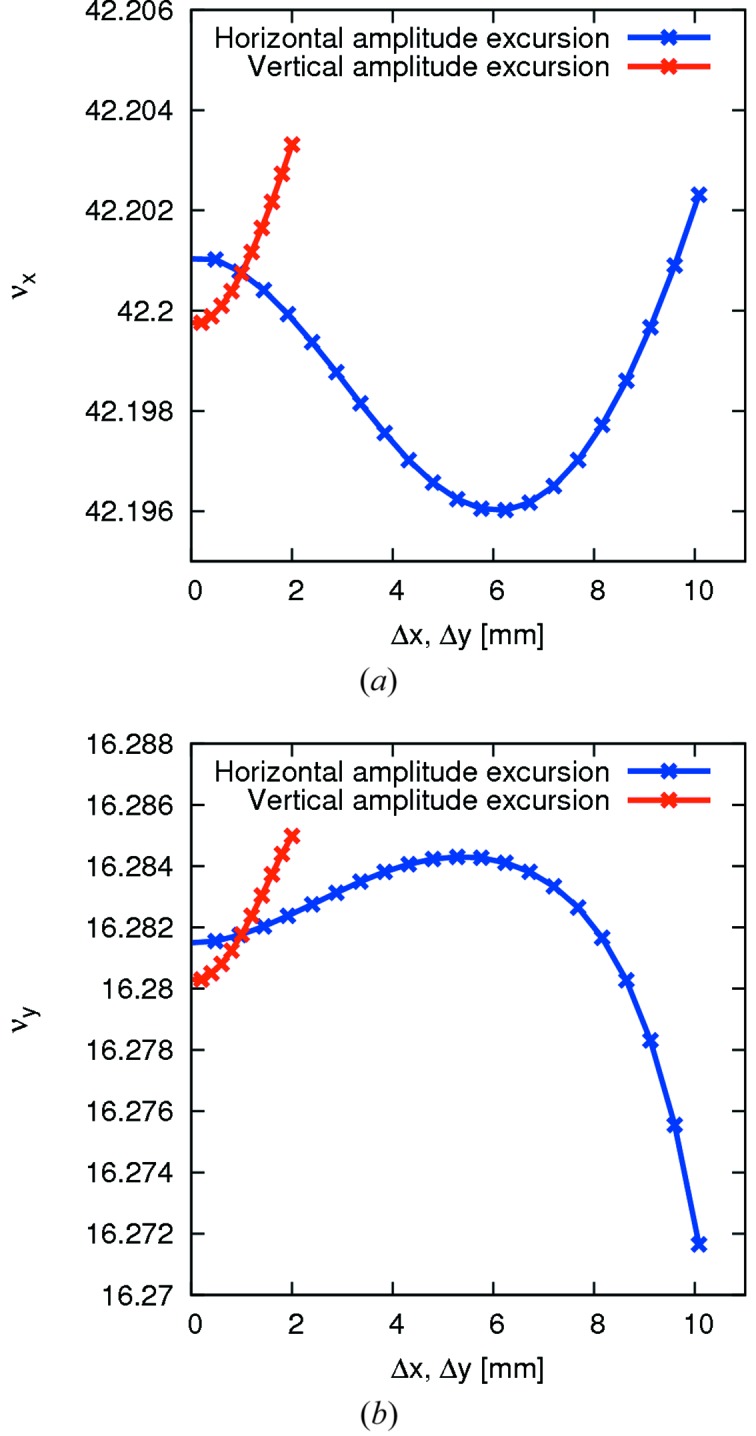
Amplitude-dependent tune shift in the MAX IV 3 GeV storage ring (Leemann, 2012*d* ▶). Note the very limited range of tune shifts that result despite amplitudes extending to the edge of the physical acceptance.

In an attempt to solve this fundamental challenge of non-linear optimization in a MBA lattice, three achromatic octupole families were introduced into the matching cells of the 3 GeV achromat in locations with appropriate β-function ratios (Leemann *et al.*, 2009 ▶; Leemann & Streun, 2011 ▶). These octupoles correct the three terms for ADTS to first order. Analogous to the linear system, which is solved to find sextupole strengths that give a certain chromaticity, a linear system can be set up to describe the ADTSs that result from an octupole in the lattice. This system can be inverted to calculate octupole strengths that give the desired ADTSs. Rather than setting the linear ADTS to zero, the octupoles in the MAX IV MBA were adjusted so the resulting overall ADTS is minimized throughout the physical acceptance (*cf.* Fig. 4 ▶).

**Figure 5:**
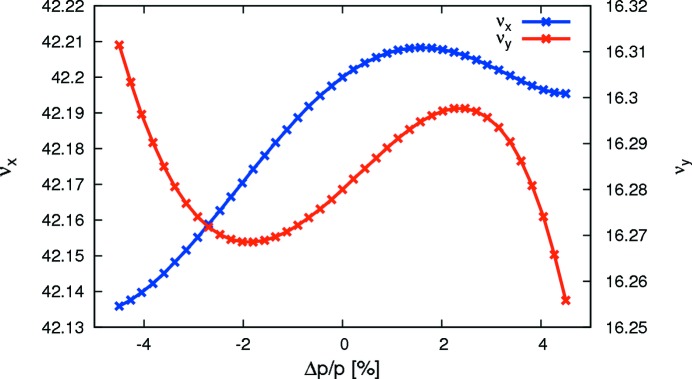
Chromaticity in the MAX IV 3 GeV storage ring (Leemann, 2012*d* ▶).

**Figure 6:**
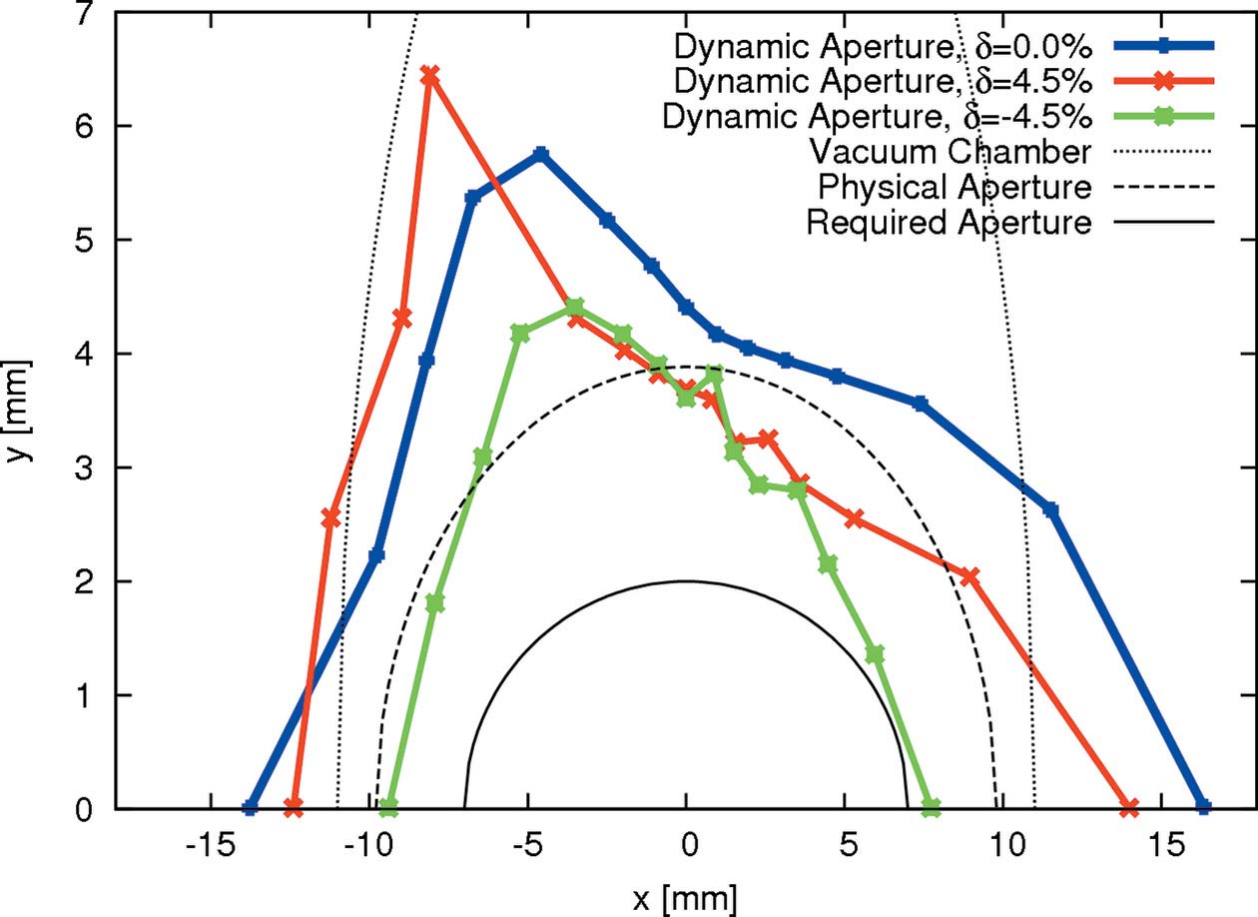
Dynamic aperture at the centre of the long straight section in the bare lattice of the MAX IV 3 GeV storage ring (Leemann, 2012*d* ▶). Tracking was performed with *Tracy-3* in 6D for one synchrotron period. For comparison, the vacuum chamber and physical aperture (projection of vacuum chamber to the track point) are also indicated in the plot.

Because the ADTSs are corrected with the octupoles, the sextupoles are freed up for first-order corrections (linear chromaticity, resonance driving terms). Some extra weight was also added to minimize second- and third-order chromaticity in an attempt to limit the chromatic tune footprint (*cf.* Fig. 5 ▶). The result of this non-linear optimization is a very limited tune footprint for particles with a range of amplitudes covering the physically accessible aperture [roughly 9 mm/2 mm (H/V) at the centre of the IDs] and energies covering the required ±4.5% acceptance. This results in large DA and MA (*cf.* Fig. 6 ▶ and §3.1), which ensure high injection efficiency and good Touschek lifetime. Frequency map analysis confirms the ‘wrap-up’ of tune shifts around the working point which results in this compact tune footprint. This holds also for a realistic machine, *i.e.* a storage ring with errors, misalignments and IDs. This shall be discussed in the next section.

### 2.3. Optics matching and orbit correction   

**Figure 7:**
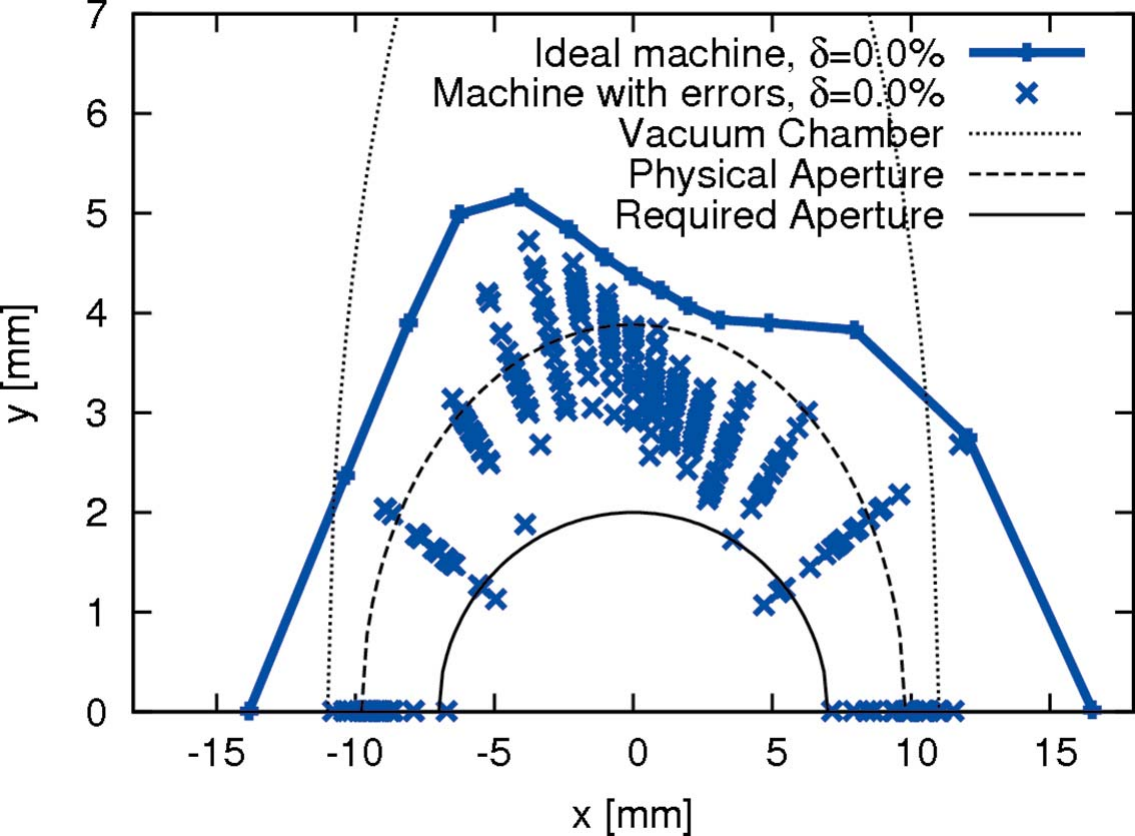
On-energy DA at the centre of the long straight section in the MAX IV 3 GeV storage ring where ten in-vacuum undulators have been added to the ring (Leemann, 2011*b* ▶). The plot shows the ideal lattice and results for 20 seeds with field and multipole errors as well as misalignments. Tracking was performed with *Tracy-3* in 6D for one synchrotron period.

With the quadrupole doublets in the matching cells the β functions in the long straights can be tuned over a fairly wide range. This allows matching of the linear optics to individual IDs. The ID matching is performed both locally (β functions are matched to minimize β beat) and globally (phase advances are corrected to restore the design working point). For the global correction the PFSs in the dipoles can be used to adjust the vertical focusing. Because this matching results in restoring the design linear optics within the achromat, the non-linear optics optimization is left almost undisturbed. If the multipolar content of the IDs is limited to specified values (Wallén & Leemann, 2011 ▶), neither sextupoles nor octupoles have to be adjusted with ID gap movement. Tracking studies with *Tracy-3* using kick maps reveal that, in the storage ring equipped with many strong in-vacuum undulators, the DA is not substantially reduced if the ID matching is properly performed. This can be recognized in Fig. 7 ▶ where ten typical in-vacuum undulators (18.5 mm period, 3.7 m magnetic length, 4.2 mm gap, 1.1 T effective magnetic field) have been added to the ring and the lattice has been matched to the IDs. The DA was calculated including various imperfections such as misalignments (of individual magnets within the magnet block as well as of the entire block), magnetic field errors and multipole errors (upright and skew multipoles).

Each achromat also contains ten horizontal and nine vertical dipole correctors as well as ten beam-position monitors (BPMs) that will be included in a slow orbit feedback. Because of the vertical beam size in the user straights reaching values as low as 2 µm r.m.s., beam stability is crucial. There are four dedicated fast corrector pairs installed around each user straight which, together with the BPM system, will allow operation of a fast orbit feedback in order to cancel beam motion effectively up to roughly 100 Hz (*cf.* §6.5).

Tracking studies have revealed that adequate DA remains when expected misalignments are added to the lattice and the orbit is corrected using the dipole correctors (Leemann, 2012*d* ▶, 2013 ▶); this also holds if multipole errors are added to all magnets (*cf*. Fig. 7 ▶). A crucial ingredient to achieving ample DA is the magnetic shunting procedure (Leemann, 2012*d* ▶). Magnets can be shunted to a common gradient within their respective families using a parallel circuit of resistor arrays. In a first stage this shunting is performed after magnet manufacturing according to magnetic field measurement results. Later this shunting can be revised according to the results of beam-based calibration measurements [*e.g.* *LOCO* (Safranek, 1997 ▶)]. In this way a low spread of magnet gradients within each family can be ensured while allowing for series connection of many magnets to one common power supply *via* a single bus.

Finally, all octupoles and sextupoles are equipped with extra windings that can be powered in various ways. This allows adding dispersive and non-dispersive skew quadrupoles for coupling control and removal of spurious vertical dispersion as well as auxiliary sextupoles in order to restore the design symmetry of the non-linear optics (Streun, 2012 ▶). These windings can also be powered as upright quadrupoles, which will be used to calibrate BPMs to the magnetic centres of the adjacent sextupoles or octupoles.

## 3. Intrabeam scattering, Touschek scattering and lifetime   

The MAX IV 3 GeV storage ring’s MBA lattice makes use of a large number of weak bending magnets which leads to low radiation losses in the dipoles compared with power radiated from insertion devices. Therefore, the ring’s zero-current emittance depends strongly on the insertion devices and gap settings (Leemann, 2014 ▶); this means the emittance during a typical user run is not necessarily constant. In addition, the large stored current along with the low emittance leads to strong intrabeam scattering (IBS) which blows up the beam’s six-dimensional emittance.

**Figure 8:**
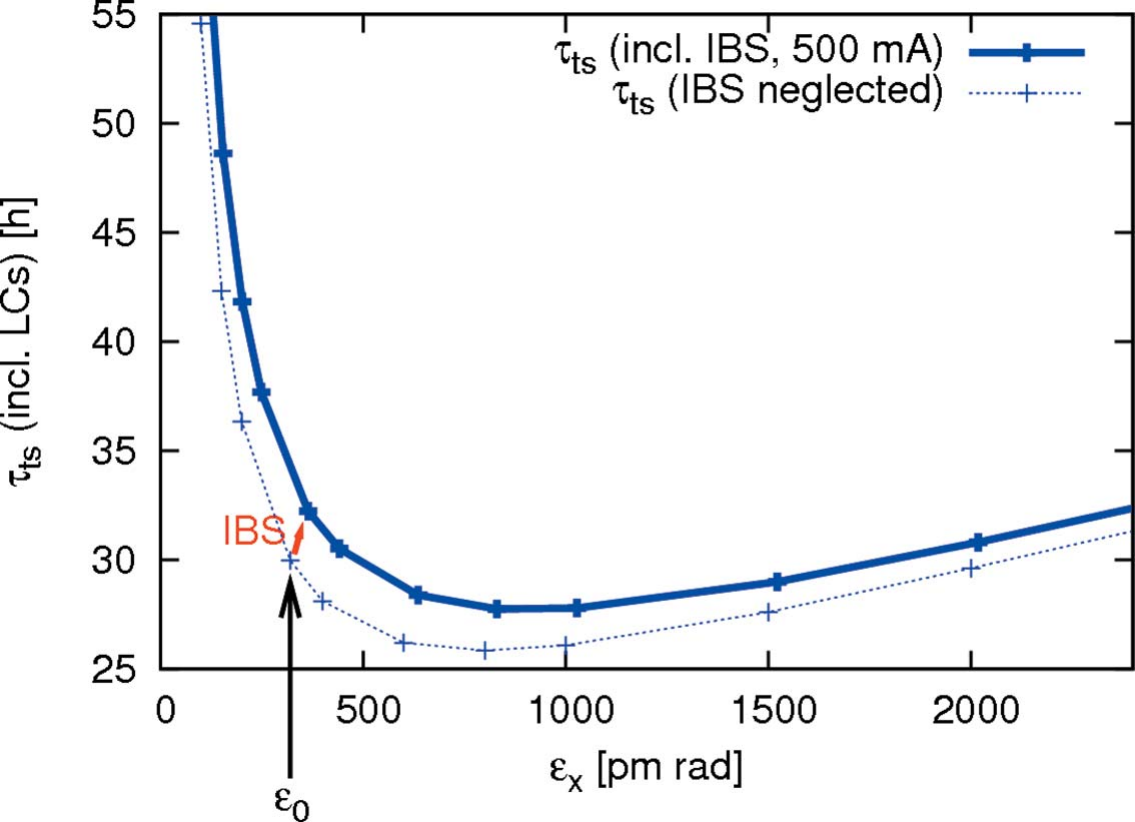
Touschek lifetime as a function of equilibrium emittance assuming the bare lattice emittance could be adjusted freely while keeping the energy spread constant (Leemann, 2012*d* ▶). The overall MA has been set to 4.5% while the vertical emittance is adjusted to 8 pm rad. The effect of Landau cavities (LCs) is included. The equilibrium emittance of the MAX IV 3 GeV storage ring bare lattice 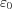 = 328 pm rad is indicated.

Touschek lifetime relies strongly on the six-dimensional emittance: it grows with increasing longitudinal emittance which makes bunch lengthening cavities attractive. On the other hand, in the ultralow-emittance regime (where transverse momenta are small compared with the large momentum acceptance), reducing the transverse emittance actually increases the Touschek lifetime (Leemann *et al.*, 2009 ▶; Leemann, 2014 ▶). This unusual behaviour in the ultralow-emittance regime is depicted in Fig. 8 ▶. Damping wigglers and insertion devices reduce the transverse emittance, but because their added losses reduce the available cavity overvoltage, they also lengthen the bunches which can increase the Touschek lifetime. Overall, Touschek lifetime will vary as a function of the resulting emittance including IBS as well as bunch lengthening.

The bare lattice has a zero-current emittance of 328 pm rad, but, at the shortest bunch length (*i.e.* at maximum cavity voltage and without Landau cavities) of 9 mm, IBS blows up the emittance by 45% for 500 mA of stored current (calculated with *ZAP* and *Tracy-3*)^3^ (Leemann, 2014 ▶). However, once the Landau cavities have been tuned in and the bunches lengthened to 54 mm (*cf*. §5.1) as expected during user operation, the IBS blow-up results in an emittance of 372 pm rad at 500 mA, *i.e.* only 13% above the zero-current emittance. For a moderately ID-equipped ring with cavities running at maximum voltage (giving an RF acceptance of 6.05%), the emittance including the effect of IBS at 500 mA and Landau cavities is expected to lie around 272 pm rad corresponding to an IBS blow-up of 16% compared with the zero-current emittance. The lowest emittance that can be expected in the 3 GeV ring should result from a ring fully equipped with IDs. In such a situation the zero-current emittance is expected to be about 187 pm rad which increases to 221 pm rad at 500 mA stored current assuming proper bunch lengthening from the Landau cavities.

### 3.1. Momentum acceptance and Touschek lifetime   

**Figure 9:**
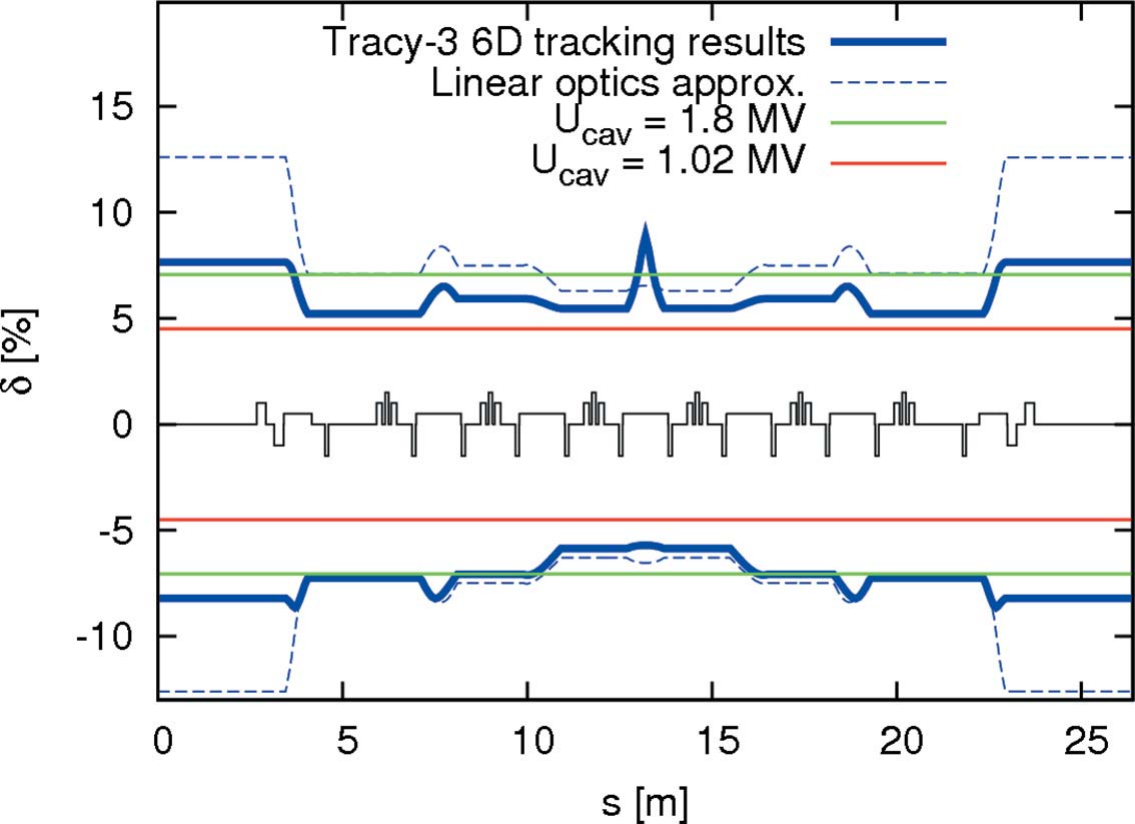
Lattice momentum acceptance for one achromat of the MAX IV 3 GeV storage ring (Leemann, 2012*d* ▶). A bare lattice with actual vacuum chamber apertures has been used. The solid blue line shows lattice MA from 6D tracking with *Tracy-3*. For comparison, the RF acceptance is shown as well: cavities at maximum voltage 1.8 MV (7.1% RF acceptance) and at 1.0 MV (4.5% RF acceptance).

The 3 GeV storage ring optics have been optimized to ensure that the MA exceeds 4.5% throughout the entire lattice in order to allow for roughly 25 h of Touschek lifetime corresponding to 10 h overall lifetime (see below). In addition to adequate off-momentum performance, this MA target requires appropriate dimensioning of the vacuum (*cf*. §6.2) and RF systems (*cf*. §6.3). The underlying assumptions for the MA and lifetime calculations are 500 mA stored current in an even fill (*i.e.* all buckets equally populated with no ion-clearing gap) resulting in 5 nC charge per bunch. The 3 GeV storage ring has six main RF cavities for a maximum overall accelerating voltage of 1.8 MV. For a bare lattice (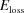 = 364 keV per turn) this corresponds to an RF acceptance of 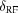 = 7.1%. The nominal inside diameter of the cylindrical vacuum chamber is 22 mm; the aperture model, however, includes additional aperture restrictions from, for example, septum and tapers. Momentum acceptance tracking in 6D with *Tracy-3* was used to verify that the resulting overall MA fulfilled design specifications (*cf*. Fig. 9 ▶). Landau cavities are expected to be tuned in during user operation. In order to include the effects of such bunch lengthening, the Touschek lifetime, however, cannot simply be scaled with the bunch length. The reason for this is IBS. When the Landau cavities are tuned in, the bunches are stretched leading not only to an increased Touschek lifetime but also to a decrease of IBS emittance blow-up. Since the resulting emittance is lowered, the Touschek lifetime is further increased (*cf*. Fig. 8 ▶) compared with the result from charge density reduction alone. Therefore, a fully self-consistent approach using 6D tracking is required (Leemann, 2014 ▶). Such studies show that even for a fully ID-equipped ring operated with Landau cavities a Touschek lifetime beyond 25 h (including the effect of imperfections and reduced vertical aperture from in-vacuum undulators) can be expected. Combined with the gas scattering lifetimes (MAX IV, 2010 ▶) this leads to an overall lifetime beyond 10 h compatible with the foreseen top-up injection scheme with one top-up injection every few minutes to ensure a top-up deadband of about 0.5%.

## 4. Injection dynamics   

The MAX IV 3 GeV storage ring will be operated in top-up mode with an overall lifetime of about 10 h. This requires top-up injections every couple of minutes. Top-up injection shots will be delivered to the storage ring from the MAX IV linac (Thorin *et al.*, 2011 ▶) serving as a full-energy injector. The maximum injection repetition rate of 10 Hz is matched to leave about seven storage ring damping times between each injection shot. A maximum of roughly 3 nC can be injected in one shot which corresponds to 0.34% of the total stored charge at 500 mA current. Assuming 10 h overall lifetime and that capture in the storage ring is highly efficient, this corresponds to a single top-up injection every other minute. If a larger top-up deadband can be tolerated, the quiet period between injection shots can be lengthened followed by several top-up shots injected at 10 Hz. Capture in the storage ring is expected to be highly efficient as a result of the low emittance of the linac combined with the comparably large acceptance of the storage ring. Bunches from the thermionic RF gun (Elafifi *et al.*, 2012 ▶) are expected to have a normalized emittance of 10 mm mrad (corresponding to horizontal and vertical emittances of 1.7 nm rad at 3 GeV) and an energy spread of the order of 0.1% r.m.s. when they are injected into the storage rings.

The original injection scheme (MAX IV, 2010 ▶) foresaw use of a closed four-kicker bump around the DC Lambertson septum in the injection straight of the storage ring. In light of the very tight beam stability requirements in the 3 GeV storage ring there was considerable doubt that four injection kickers could be aligned, balanced and synchronized well enough to prevent perturbation of the stored beam beyond the limits of these stability requirements. Furthermore, since the injection bump would have contained several strong sextupoles and octupoles, the bump could not be closed properly for all amplitudes and all particles in the bunch. This led to the development of a new injection scheme for the MAX IV storage rings.

### 4.1. Pulsed multipole injection   

Intrigued by KEK’s pioneering work on pulsed quadrupole (Harada *et al.*, 2007 ▶) and pulsed sextupole injection (Takaki *et al.*, 2010 ▶), it was recognized that a pulsed multipole had the potential to make top-up injection shots into the MAX IV storage rings transparent to users while allowing for a substantial reduction of complexity. Instead of four dipole kickers and their pulsers, only a single magnet and its pulser were required; the pulsed magnet could be aligned to the stored beam through beam-based measurements.

**Figure 10:**
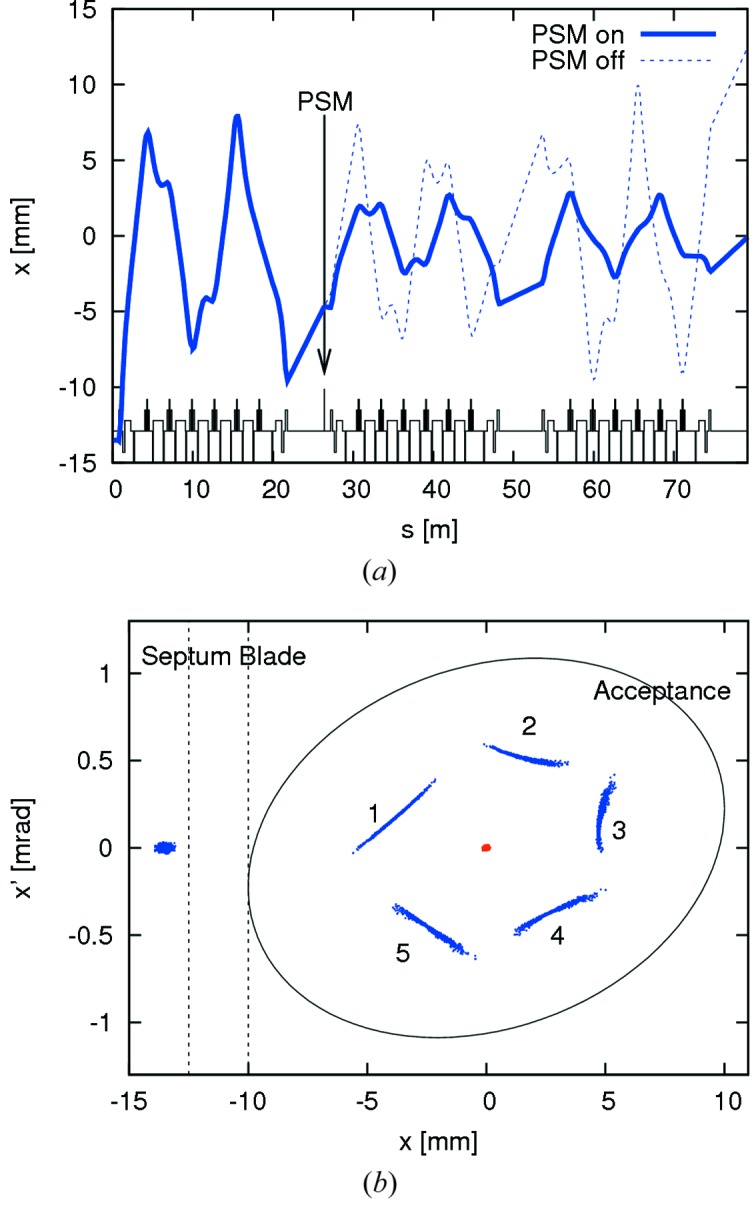
Top: trajectory of the injected bunches in the MAX IV 3 GeV storage ring starting at the septum (Leemann, 2012*b* ▶). The PSM is installed in the second long straight and kicks the injected bunch into the ring acceptance. Bottom: tracking of injection and capture of 1000 particles at the septum in the 3 GeV storage ring. The first five turns are indicated in blue. For comparison, the stored beam is indicated in red.

Because of the low emittance of the injected bunches, sampling the gradient of a multipole at injection was not considered to be a problem. The strong non-linearity of betatron motion in the 3 GeV storage ring, however, required optimization of the pulsed multipole injection (PMI) scheme with tracking studies in order to determine the best location and kick amplitude for the pulsed multipole (Leemann, 2012*b* ▶). A scheme was developed for a pulsed sextupole magnet (PSM) using both single-turn and two-turn injection as well as different kick strengths (*cf*. Fig. 10 ▶). It was shown that such an injection scheme did indeed allow for very high capture efficiency of the injected bunch with only minute perturbation of the stored beam. Following the KEK PSM, a solid-iron sextupole was designed for injection in MAX IV. Despite the smaller magnet apertures in MAX IV and the corresponding reduction of the magnet inductance compared with the KEK PSM, the required voltages remained very high as a consequence of the short pulse duration (Leemann & Dallin, 2013 ▶).

**Figure 11:**
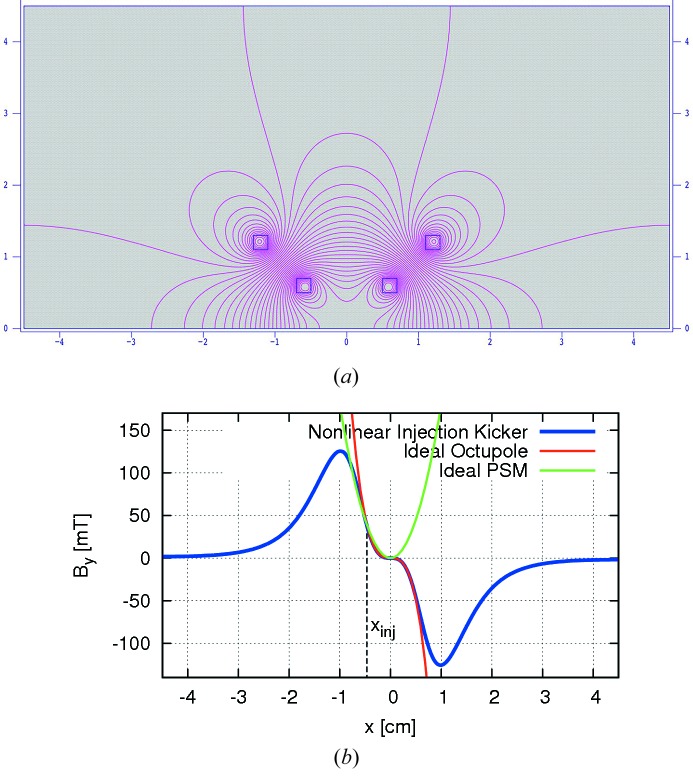
Upper half geometry (top) and resulting field profile in the midplane (bottom) of a BESSY-type non-linear injection kicker adapted to the MAX IV 3 GeV storage ring (Leemann & Dallin, 2013 ▶). Note that the resulting field around the centre is similar to an octupole.

**Figure 12:**
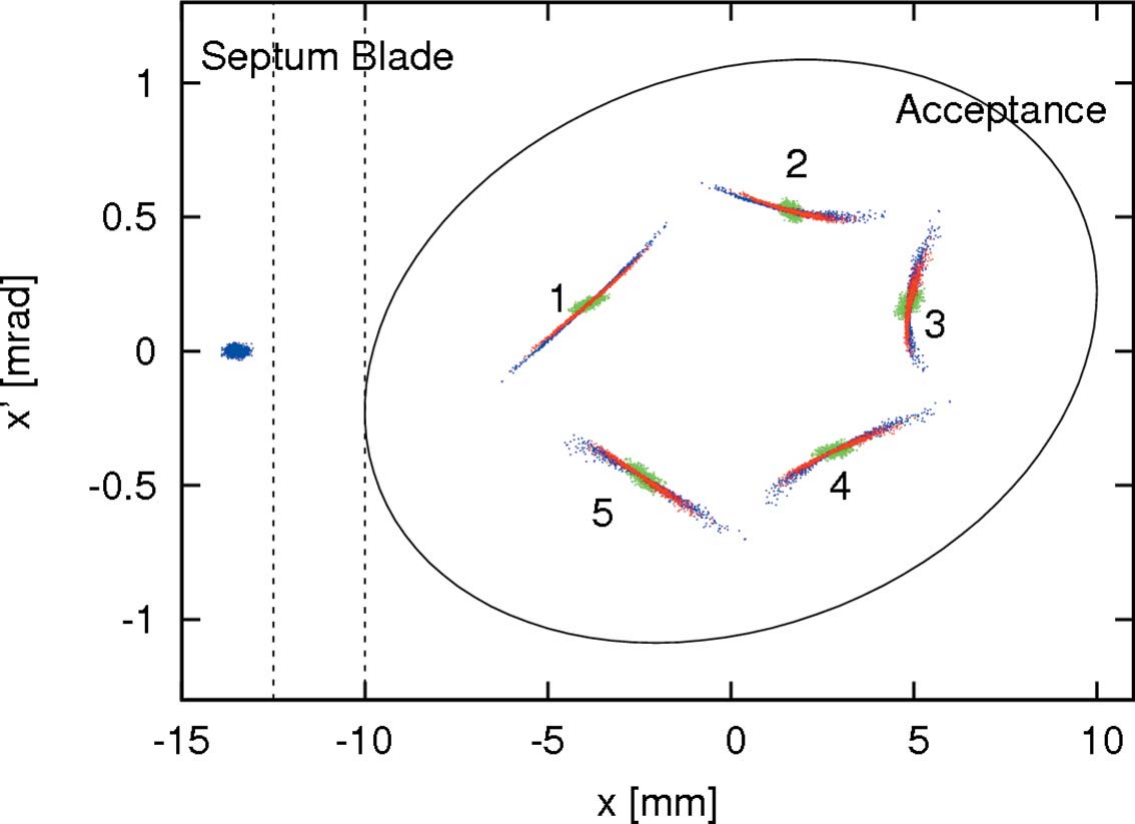
Tracking of 1000 particles of an injected bunch during the first five turns in the MAX IV 3 GeV storage ring using single-turn injection (Leemann & Dallin, 2013 ▶). Injection with the BESSY-type kicker (blue) is compared with a PSM (red). For reference, a pure dipole kick (green) has also been included.

A novel non-linear injection kicker developed for BESSY II (Atkinson *et al.*, 2011 ▶) appeared to solve these issues. Its stripline-like design generates a non-linear magnetic field with limited inductance. The non-linear field profile crosses zero at the centre, is flat around this area, and achieves a highly localized maximum several millimetres from the centre. In this way a large kick can be supplied to the injected bunch while minimizing any residual kick to the stored beam. In the vicinity of the stored beam the field is octupolar which leads to an even lower perturbation than a PSM. At BESSY II this kicker delivers roughly 1 mrad of kick at 12 mm distance from the stored beam. The separation of the injected bunch from the stored beam in MAX IV is, however, only about 5 mm (Leemann, 2012*b* ▶). In order to apply the BESSY kicker design to MAX IV the vertical separation of the inner rods would have to be reduced to levels that are no longer compatible with the vertical acceptance requirements. However, because the injected beam at MAX IV has such a low emittance, bunches can be injected on the slope of the kicker field. Fig. 11 ▶ shows the geometry of a BESSY-type non-linear injection kicker adapted to MAX IV along with the generated field profile. Tracking of injection and capture of bunches in MAX IV using a BESSY-type kicker is displayed in Fig. 12 ▶. For comparison, the effect of a PSM and a pure dipole are included. The latter clearly disperses the injected beam less than the two multipole kickers; however, since it also kicks the stored beam it is not compatible with the requirement of transparent top-up injection. Tracking studies have also shown that the perturbation of the stored beam by a BESSY-type kicker including a vacuum chamber with a thin metallic coating remains negligible if the kicker is aligned properly to the stored beam. A collaboration between MAX IV, SOLEIL and BESSY has been launched with the goal of developing and manufacturing BESSY-type kickers for both MAX IV storage rings and SOLEIL.

### 4.2. Injection with a single dipole kicker   

Commissioning an entirely new storage ring relying only on PMI is demanding. The kick received by the injected bunch depends heavily on the bunch position and angle at the injection septum as well as the optics between the septum and the injection kicker. In a new storage ring these parameters are not known to high accuracy and optics deviations from design are to be expected as a consequence of misalignments, calibration uncertainties and/or cabling errors. Such errors can be diagnosed and resolved; however, usually beam-based measurements are employed. Hence, a minimum amount of injection and capture have to be ensured so commissioning can progress.

**Figure 13:**
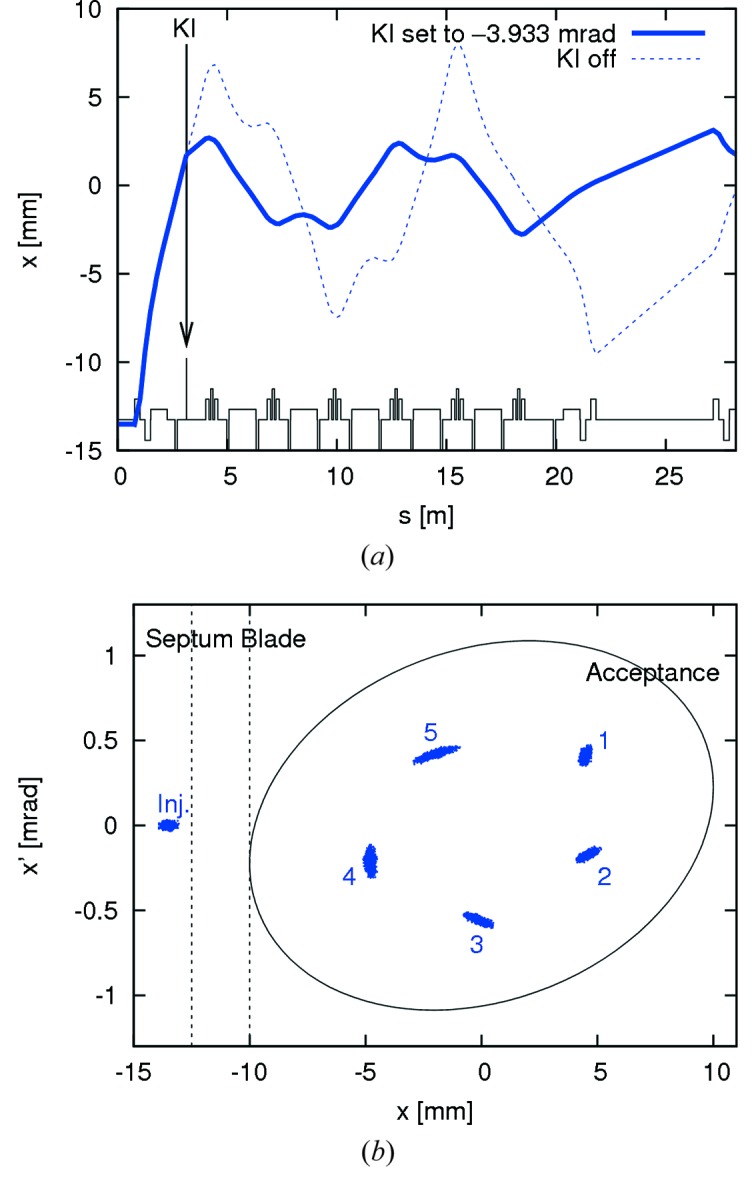
Top: off-axis injection with a single dipole kicker (KI) in the MAX IV 3 GeV storage ring starting at the septum (Leemann, 2012*a* ▶). Bottom: phase space plot showing tracking of injection and capture (first five turns are indicated) of 1000 particles at the septum.

**Figure 14:**
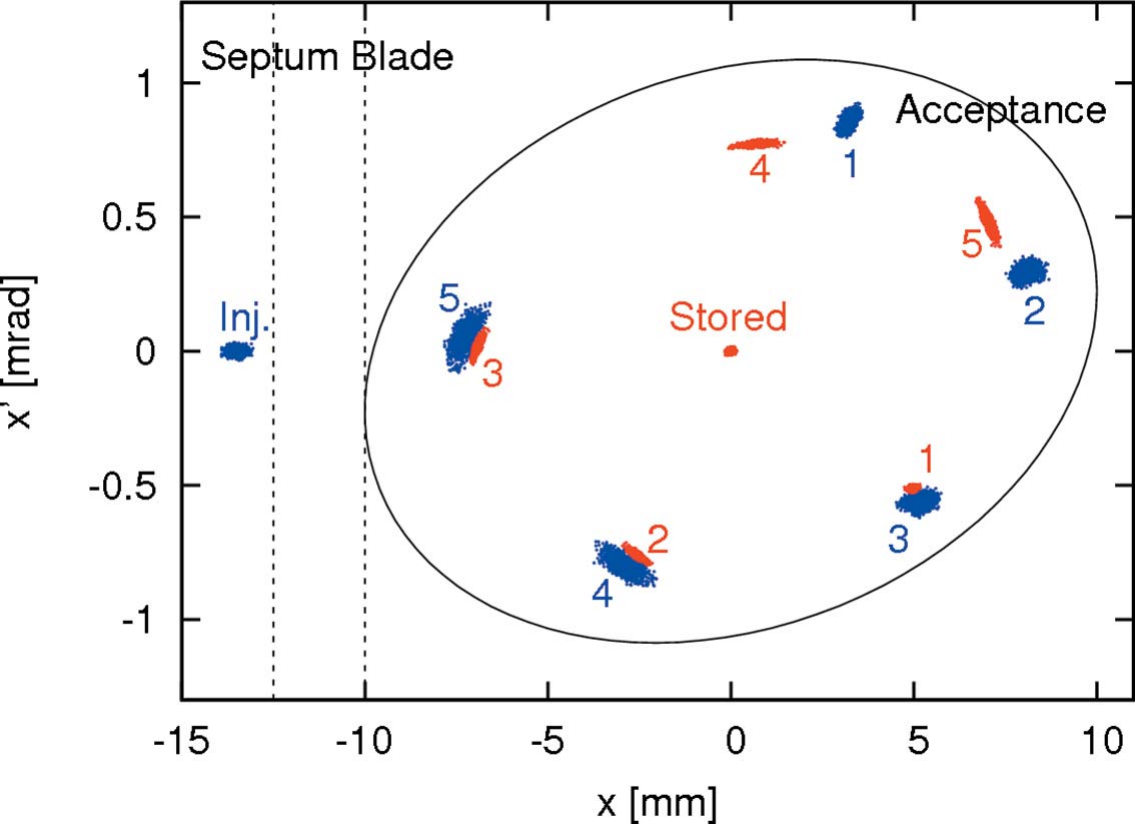
Tracking of 1000 particles of a stored bunch (red) and 1000 particles of an injected bunch (blue) in the MAX IV 3 GeV storage ring (Leemann, 2012*a* ▶). The dipole kick strength has been reduced to allow for stacking: the injected bunch is captured while already stored charge in the same bucket is not ejected out of the machine acceptance. The first five turns after injection are indicated.

In order to provide a simple and robust injection into the storage ring to allow for early commissioning activities, an alternative to PMI was desired. Injection with a single dipole kicker presented a solution (Leemann, 2012*a* ▶). Although a dipole kicker does not allow for transparent top-up injection, it offers an injection with little dependence on initial parameters and optics. In the MAX IV 3 GeV storage ring, a horizontal dipole kicker installed in the first short straight after the injection septum allows for both on- and off-axis injection (*cf*. Fig. 13 ▶).^4^ In fact, if the kick strength of the dipole kicker is reduced, the injection kick can be divided between the injected bunch and any stored charge already in the bucket so as to allow for some stacking (*cf*. Fig. 14 ▶).

Once small amounts of beam are injected and stored in the storage ring, beam-based measurements will allow the optics to be adjusted to its design values. At this point commissioning of the pulsed multipole magnet can begin. Pulsed multipole injection will then allow accumulation of large amounts of charge in the storage ring without perturbation of already stored beam thus enabling transparent top-up operation for users. The single dipole kicker will from then on serve as a horizontal pinger magnet for machine studies.

## 5. Coherent collective instabilities   

The MAX IV design concept leads to several challenges in reaching stable operation of the machine at high currents. These challenges arise from the compact magnet design, which calls for a small vacuum chamber aperture leading to an increased interaction of the beam with its environment; in particular, the resistive wall impedance, which scales inversely as the third power of the beam pipe aperture, may lead to the excitation of transverse coupled-bunch modes. Moreover, the low-emittance lattice design leads to a small dispersion function in the arcs (8 cm maximum), which, coupled with the large bending radius, results in a small momentum compaction factor, and consequently small values for single-bunch instability thresholds, particularly for the microwave instability and transverse mode coupling instability.

In order to face the issues described above, the MAX IV facility design relies on the fact that short light pulses will be produced by a linac source (the 3 GeV injector linac and corresponding short-pulse facility), which relieves the storage rings from the need to achieve short-bunch and single-bunch high-current operation. This allows us to:

(i) Choose a relatively low RF frequency for the accelerating cavities, which leads to longer bunches for a given RF momentum acceptance.

(ii) Use harmonic (also called Landau) cavities (HCs) operating in passive mode in order to further elongate the bunches, reducing the charge density thus alleviating intrabeam scattering, which is not only essential to reach the target equilibrium emittances at high current but also increases the average current thresholds for longitudinal single-bunch fast instabilities and reduces the beam-induced heat load on vacuum chamber components. In addition, the Landau cavities provide increased tune spread that helps prevent instabilities and makes up for the relatively long radiation damping times that result from the large bending radius.

(iii) Require only multibunch operation with relatively low current per bunch.

### 5.1. Harmonic cavities and bunch lengthening   

As noted above, long bunches and HCs are an essential ingredient in the MAX IV design concept. Practical experience (Georgsson *et al.*, 1998 ▶) has demonstrated the possibility of operating such cavities passively, so that the beam itself provides the excitation of the HC. If one chooses the HC parameters (Hofmann & Myers, 1980 ▶) such that the first and second derivatives of the total voltage seen by the beam are zero at the synchronous phase, the longitudinal potential well that holds the bunches becomes approximately quartic and the incoherent synchrotron frequency grows approximately linearly with amplitude for small amplitudes, being exactly zero at the centre of the bunches. For the MAX IV 3 GeV ring at 500 mA nominal beam current, with an accelerating voltage of 1.6 MV and 856 keV energy loss to synchrotron radiation per turn, such *flat potential* conditions correspond to a HC shunt impedance of 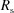 = 2.017 MΩ and a HC detuning 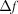 = −28.43 kHz, which leads to an r.m.s. bunch length of 54.1 mm, whereas the natural bunch length without HCs is 10.1 mm. This bunch lengthening is accompanied by a widening of the synchrotron frequency distribution and a corresponding increase of the Landau damping rate of coherent modes.

Even though this choice of parameters for the HC system leads to flat symmetric bunch shapes, one must keep in mind that passive operation of HCs always implies operation on the Robinson unstable slope of the fundamental mode of the HCs; in other words, one must rely on other damping mechanisms such as synchrotron radiation damping and Robinson damping from the fundamental mode of the main (100 MHz) RF cavities to keep the beam stable. In fact, for the MAX IV 3 GeV ring parameters mentioned above, the Robinson growth rate from the fundamental mode of the HC at flat potential conditions is too large (67 s^−1^) to be compensated by radiation damping alone (39 s^−1^).

**Figure 15:**
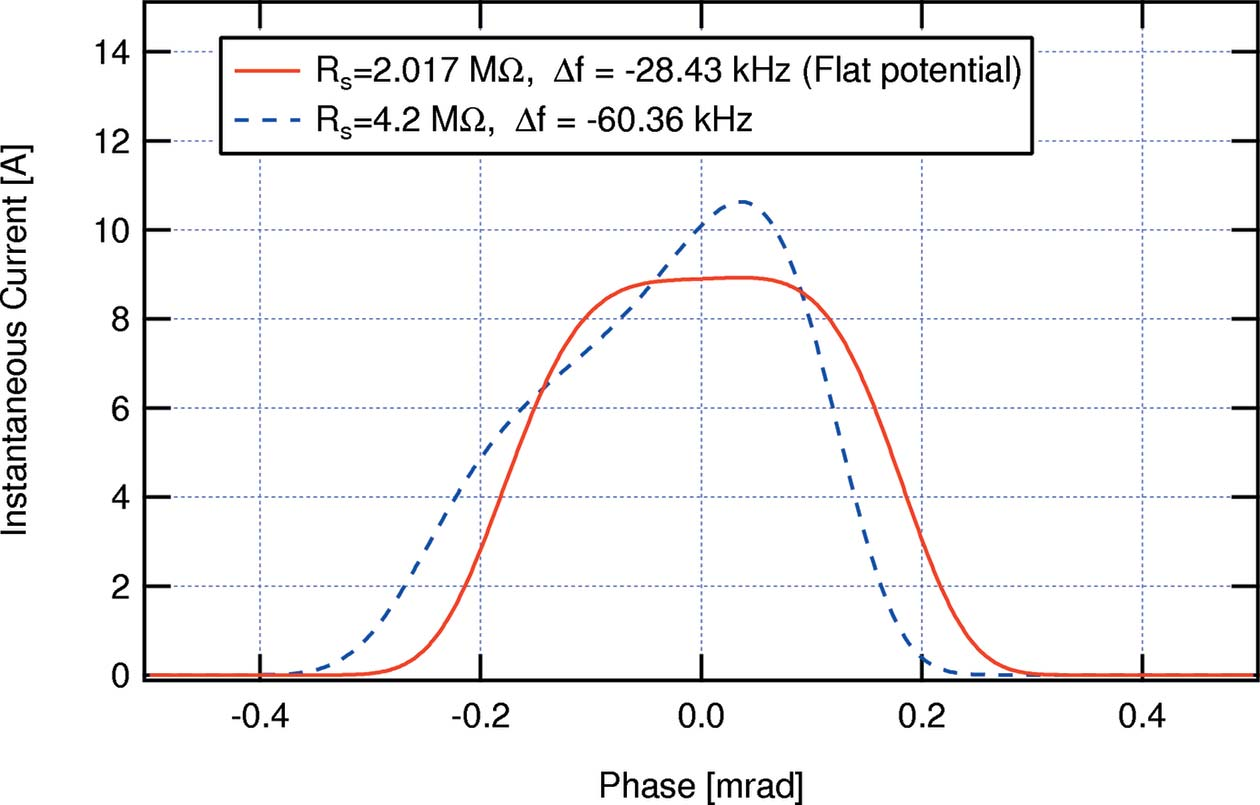
Equilibrium longitudinal bunch density distribution for two different settings of the HC system in the MAX IV 3 GeV ring.

However, calculations for the MAX IV parameters (Tavares *et al.*, 2013 ▶, 2014 ▶) indicate that lengthening similar to the flat potential case can also be achieved with a passively operated HC with higher detuning and correspondingly lower Robinson anti-damping, as long as the HC shunt impedance can be raised significantly above the flat potential conditions. By choosing, for example, a shunt impedance 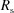 = 4.2 MΩ and 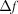 = −60.36 kHz (Fig. 15 ▶), we can reach an r.m.s. bunch length of 54.2 mm and the Robinson growth rate due to the HC fundamental mode is then reduced by more than a factor of four to 15.3 s^−1^, well within the range of radiation damping. The incoherent synchrotron tune spread can also be made similar to or even larger than the spread corresponding to the flat potential case, as long as there is enough margin in HC shunt impedance, even though the bunches become somewhat asymmetric and show a slightly larger peak current. This is in fact the approach adopted for the MAX IV 3 GeV ring, where a significant margin in shunt impedance above the flat potential condition is provided by installing three identical HCs, each with a shunt impedance of 2.5 MΩ. Having the total shunt impedance split among three different cavities allows tailoring the actual shunt impedance seen by the beam by tuning each cavity independently and additionally also permits the power dissipated in each cavity to be kept within acceptable levels.

### 5.2. Multibunch instabilities   

**Figure 16:**
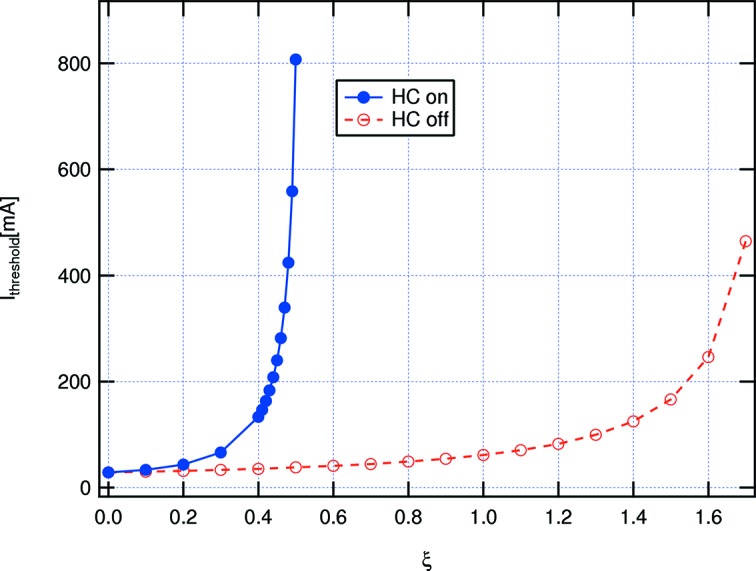
Threshold current at which the growth rate of the fastest growing coupled bunch mode (and lowest synchrotron mode number, *i.e.* the rigid bunch mode) driven by the resistive-wall impedance equals the transverse synchrotron radiation damping rate as a function of chromaticity for two different bunch lengths corresponding to the situations with and without HCs.

Transverse coupled-bunch modes driven by the long-range resistive wall wakefields are a potential concern given the small pipe radius (11 mm) in the MAX IV 3 GeV ring. Lengthening of the bunches provides us, however, with a very efficient means of enhancing the effectiveness of positive chromaticity in fighting these unstable modes. In fact, the long bunches imply a narrow frequency span of the head–tail modes, which leads to a small overlap of the chromaticity-shifted eigenmode spectra with the resistive wall impedance spectrum, which is concentrated at low frequencies leading to lower growth rates. This expectation is confirmed by applying the standard Sacherer formalism as implemented in the computer code *ZAP* (Zisman *et al.*, 1986 ▶) to calculate the corresponding growth rates as shown in Fig. 16 ▶. The same trend is also confirmed by a more direct frequency domain calculation with the code *rwmbi*, in which actual eigenvectors (instead of the approximate Hermitian modes adequate for Gaussian bunches) are used (Tavares *et al.*, 2011 ▶).

Even though these results are reassuring, the direct use of the Sacherer formalism for the situation with HCs might be questioned, as the very concept of synchrotron modes seems to lose its validity in that limit since, for flat potential conditions, the phase focusing at the very centre of the bunches vanishes. However, particle tracking simulations (Klein *et al.*, 2014 ▶) give indications that the lengthened bunches are indeed safe from resistive-wall-driven instabilities at nominal current levels.

Longitudinal multibunch instabilities are driven by high-Q trapped modes in vacuum chamber components as well as by the HOMs in RF cavities. While the growth rates of unstable modes can be calculated from the standard Sacherer formalism when no HCs are present, a modified version of the theory (Bosch *et al.*, 2001 ▶) is required to predict the growth rates when the HCs flatten out the longitudinal potential well. An analysis based on this formalism has been carried out for MAX IV making use of a detailed longitudinal impedance budget (Günzel, 2009 ▶) and led to the conclusion that the ratio between the allowed shunt impedance (*i.e.* the one below which the beam is stable) and the actual estimated shunt impedance for all modes trapped in the chamber is quite large for the vast majority of components, going down to about 3 for a resonance at the double flanges around the BPM bodies. Finally, the power deposited on vacuum chambers by beam-induced fields is found to be negligible as long as the bunches are lengthened by the HCs.

### 5.3. Single-bunch instabilities   

In the MAX IV 3 GeV ring, longitudinal single-bunch instabilities are a source of concern not because of the associated increase in bunch length (since by design we lengthen the bunches anyway) but rather due to the potential increase in energy spread associated with the microwave instability and the resulting degradation of the undulator spectra.

Longitudinal single-bunch instabilities have been studied by multi-particle tracking (Klein *et al.*, 2013 ▶). In those studies, a longitudinal impedance model composed of seven resonators as well as purely resistive and inductive components was fitted to the numerically determined impedance of the vacuum chamber components (Günzel, 2009 ▶). The effects of the passively operated HC were included in the code *mbtrack* and the results indicate (Klein & Nagaoka, 2013 ▶) that the beam remains stable without significant increase of the energy spread at the nominal beam current of 2.84 mA per bunch. These studies have also allowed the identification of a few components, in particular bellows and BPMs, as the main items responsible for determining the instability thresholds.

While one may expect that the use of HCs can improve the situation for fast longitudinal instabilities, since the lengthening of the bunches reduces the bunch peak current for a given stored average current, this may not necessarily happen for fast transverse single-bunch instabilities. Preliminary calculations (Tavares *et al.*, 2011 ▶) with the computer code *MOSES* (Chin, 1988 ▶) based on a simplified single-resonator impedance model indicated that a relatively low chromaticity of 0.5 was enough to keep the beam stable against fast transverse instabilities at the nominal beam current. More detailed tracking studies based on a numerically determined transverse impedance budget and subsequent impedance modelling studies for the transverse plane are ongoing (Klein *et al.*, 2014 ▶) and have so far confirmed the same trends observed in the simplified models.

## 6. Engineering and instrumentation   

### 6.1. Magnets   

The main requirement for the 3 GeV ring magnets^5^ is to produce the large integrated focusing strengths needed to achieve ultralow emittance with relatively short magnets so as to minimize the total machine circumference and associated costs. Additional objectives include tight alignment tolerances, low sensitivity to vibrations and an integrated design concept that allows for streamlined installation and system tests.

The key ingredient to achieve these goals is a reduced magnet gap: 25 mm bore diameter in quadrupoles and sextupoles and 28 mm pole gap at the transverse centre of the gradient dipoles. A small pole aperture allows realisation of a compact lattice for several reasons: first, the lengths of the elements can be made shorter for a given integrated strength while keeping the pole-tip fields below saturation levels; second, the distance between consecutive magnetic elements is constrained to about one pole gap by the need to limit field quality deterioration caused by fringe-field effects, and, third, a reduced pole gap allows for smaller coil cross-sections which makes it easier to fit the coil ends between magnets.

The requirements on low sensitivity to vibrations and tight alignment tolerances are achieved by having all magnets in each cell built as a single unit, *i.e.* the magnet block. The dipole poles and quadrupole pole roots are machined out of two yoke halves that serve also as a girder in which all magnets in the cell are assembled. The magnet blocks are then supported by massive concrete stands. With this concept the relatively small and light magnet blocks have high natural vibration frequencies, which makes them insensitive to typical floor vibrations. Moreover, the relative alignment of the various magnets within a block is defined by the combination of machining accuracy of the yokes and poles and the corresponding assembly errors, which can be performed to tighter accuracies (±20 µm) than optical alignment of the whole block. In this way, resulting misalignments of individual magnets tend to be correlated throughout a magnet block leading to partial compensation among the resulting kicks. Last but not least, the integrated magnet design concept assumes that the magnetic field quality is determined by mechanical tolerances so that no further adjustment based on magnetic measurements (such as, for example, shimming for alignment to magnetic centres) is planned, except for possible shunting for the strength of the main components. This simplifies considerably the installation and system tests procedure as well as contributes to cost reduction.

### 6.2. Vacuum   

The vacuum design^6^ is defined by the small magnet apertures which lead to narrow low-conductance vacuum chambers (22 mm inner diameter). The major challenges of the design are therefore the need to reduce photodesorption and provide adequate pumping all along the chambers as well as the safe extraction of the heat load from synchrotron radiation on the chamber walls. In order to face these issues, the chamber consists of a cylindrical copper tube which is coated with non-evaporable getter (NEG) alloy. Even though the NEG coating technology, originally developed at CERN (Benvenuti *et al.*, 2001 ▶), has already been applied on a large scale to insertion device chambers (Kersevan & Hahn, 2006 ▶) as well as to straight vessels in synchrotron radiation sources [about 56% of the SOLEIL chambers are NEG coated (Herbeaux *et al.*, 1998 ▶)], the MAX IV 3 GeV storage ring will be the first light-source storage ring to be close to 100% NEG coated, including straight sections as well as dipole (bent) chambers. The coating of such narrow tubes, particularly the chambers which are used for extraction of the insertion device radiation, presents a significant engineering challenge. Some of the issues that were faced during the development phase of NEG coating procedures for the MAX IV chambers (Calatroni *et al.*, 2013 ▶) include the coating of bent chambers, already demonstrated for larger diameters at MAX II (Hansson *et al.*, 2010 ▶), the coating of wire-cut parts and of chambers made of different materials such as copper and stainless steel brazed together.

Cooling of the chambers is realised by means of electron-beam welded cooling channels along the chambers, which act as distributed heat absorbers. Finite-element analysis has been extensively used (Al-Dmour *et al.*, 2011 ▶) to understand the effects of the deposited heat and ensuing stresses on the mechanical integrity of the cambers as well as on the positional stability of the BPM blocks, a critical issue due to the tight requirements on beam position stability that result from the machine’s ultralow emittance.

As a result of the small clearance between magnets and chambers, the chamber assembly and activation of full achromats (approximately 22 m length) will not be performed *in situ*, but rather in a bakeout oven assembled on a table over the lower yoke halves. The fully assembled and activated achromat chamber will then be lowered onto the magnet halves and the upper yoke halves assembled on top.

### 6.3. RF system   

A main RF system of relatively low frequency, 100 MHz, has been chosen. The main cavities are made entirely of copper and are of normal-conducting capacity-loaded type, where the present cavities of the MAX II and MAX III storage rings have served as prototypes (Andersson *et al.*, 2002 ▶, 2011 ▶; MAX IV, 2010 ▶). The shunt impedance amounts to 1.6 MΩ . It is not possible to reach as high a shunt impedance per metre cavity length as for higher-frequency systems. However, a low-frequency system requires a lower overvoltage (peak voltage to synchronous voltage ratio) for a given RF energy acceptance. In the end, for the MAX IV case, a low-frequency system turns out to be advantageous considering power consumption. As can be seen from Table 2 ▶, for a fully ID-equipped MAX IV 3 GeV ring with 4.5% RF energy acceptance the total copper losses will amount to 185 kW. A 500 MHz system occupying the same accelerator length and providing the same RF energy acceptance would generate roughly 400 kW in copper losses. High-efficiency (60–70%) RF transmitters available at 100 MHz additionally help in power saving.

From a beam dynamics point of view, it may not be obvious at first sight why a low-frequency choice should be preferred. In fact, the resulting lower RF voltage leading to the same RF energy acceptance for the low-frequency choice still results in a higher bunch peak current for the same total stored beam current, even though the bunches are longer. Again comparing with a 500 MHz system, the bunch peak current would be roughly 35% higher. However, when HCs are used to lengthen the bunches, as described in §5, the impact of frequency choice on the bunch peak current is reduced. In fact, if we excite the HCs so that the flat potential case is reached, the bunch peak currents for 100 MHz and 500 MHz RF systems differ by only about 15%.

Moreover, while the peak bunch current is relevant for single-bunch instabilities, the growth rate of coupled-bunch instabilities depends on the total circulating current and on the overlap of the impedance with the bunch spectrum. As a result, choosing a low RF frequency while keeping the total current constant reduces the growth rates of coupled-bunch instabilities due to the longer bunches.

The HCs are chosen to be of similar type as the main cavities, primarily because the capacity loaded type has the advantage of pushing higher-order modes to relatively high frequencies compared with pillbox cavities. The fundamental mode shunt impedance per cavity stays at the moderate value of 2.5 MΩ. Table 2 ▶ shows the relevant numbers for the RF parameters for a plausible commissioning case and for a fully ID equipped MAX IV 3 GeV ring, both with 4.5% RF energy acceptance. The final design value for the RF station power has consequently been set at 120 kW. To reach this power, two 60 kW transmitters are combined.

### 6.4. Diagnostics   

#### 6.4.1. General   

Both storage rings will be equipped with a set of standard diagnostic equipment. This includes scraper sets to allow measurement of vacuum lifetimes, stripline antennas for tune excitation and a fluorescent screen profile monitor directly after the injection septum to better diagnose the injection process. The latter will be complemented with an extra BPM.

#### 6.4.2. BPMs   

The MAX IV storage rings will use standard capacitive button BPMs. In the 3 GeV ring the standard BPM housing will have a circular aperture of 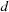 = 25 mm. A few non-standards exist in regions where a larger horizontal aperture is required for injection.

The capacitive buttons are based on the ALBA design (Olmos *et al.*, 2006 ▶) for both rings but with somewhat reduced dimensions. Altogether the BPM designs have sensitivities, *i.e.* changes in the 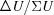 signal due to beam movement, of 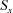 = 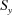 = 11% mm^−1^ for the 3 GeV ring standard units.

All BPMs will be equipped with Libera^®^ Brilliance+ electronics. Apart from allowing the now standard applications of fast orbit feedback and *LOCO* response matrix analysis (Safranek, 1997 ▶) to calibrate the linear optics, the BPMs will also have single-turn and turn-by-turn data acquisition capability. The former will assist in first-turn beam threading while the latter will provide, amongst others, the possibility of investigating the resonance driving terms using frequency map analysis (Robin *et al.*, 2000 ▶). Pinger magnets will be installed for both planes in order to induce transient betatron oscillations. The eventual aim of such analysis is of course correcting errors in the non-linear optics (Bartolini & Schmidt, 2005 ▶).

The 3 GeV storage ring will be equipped with 200 BPMs. Given the betatron tunes of 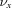 = 42.2, 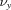 = 16.28, the betatron period is thus well sampled. It should be noted that the large number of BPMs in the 3 GeV storage ring was motivated primarily by the need to restrict orbit excursions inside the strong sextupoles, which can otherwise result in an increased emittance coupling between the transverse planes.

#### 6.4.3. Transverse emittance diagnostics   

Each ring in the MAX IV project will be equipped with two diagnostic beamlines based on imaging of the electron beam using visible to ultraviolet synchrotron radiation. The design will be similar to that given by Andersson *et al.* (2008 ▶) and Saá Hernández *et al.* (2013 ▶), where π-polarized light plays an important role in determining the vertical emittance (Andersson *et al.*, 1996 ▶). The method relies on an accurate determination of the transverse beam sizes, utilizing the wave properties of the emitted synchrotron radiation (Chubar & Elleaume, 1998 ▶), both in the vertical and horizontal directions (MAX IV, 2010 ▶). The beamlines are placed so that both a low and a high dispersion point in the lattice are observed. In this way it becomes possible to determine both horizontal and vertical beam emittance as well as energy spread. Also, the horizontal and vertical dispersion will be directly measured at the source point. Only the betatron values need to be determined from lattice fits to the orbit response matrix, which should be accurate to within a few percent. The most demanding beam size measurements are at the 3 GeV ring, where we expect to be able to determine vertical/horizontal beam sizes of 6 µm/18 µm with an r.m.s. uncertainty of 0.3/1.0 µm. The derived vertical/horizontal emittance in the vicinity of 2 pm rad/200 pm rad will have a relative r.m.s. uncertainty of roughly 10%/15%.

### 6.5. Orbit feedback   

The orbit feedback systems in both storage rings share the same conceptual design (Sjöström *et al.*, 2011 ▶) and differ mainly in two areas: number of sensors and actuators, as well as the physical design of the BPMs and dipole corrector magnets. Electronics, power supplies, algorithms, software and general topology will be identical. In terms of required orbit stability the 3 GeV ring is the more demanding with vertical beam sizes of 2–4 µm on the long straights, which results in orbit stability requirements of the order of 200 nm. The orbit feedback system uses two separate global feedback loops, which use the same set of BPM sensors but two different sets of actuators.

The ‘slow’ loop will handle corrections for slow drifts. Update frequencies are expected to be in the 0–10 Hz region. Actuators will be dipole corrector magnets with solid iron yokes, which in the 3 GeV ring are located around Cu vacuum chambers. Their expected bandwidth will be around 30 Hz (−6 dB point) and have a kick strength of 0.35–0.42 mrad, depending on location. Given the long time period between updates it is possible to rely on the main control system for data gathering, calculations and transmission of new set points to the actuators. The loop bandwidth, *i.e.* the region in which there is noise attenuation, is expected to be roughly 100 times lower than the loop update frequency.

The ‘fast’ loop will handle orbit noise and transient disturbances. The update frequency of both sensor data and actuator set values will be 10 kHz. While the dipole corrector magnets that will be used as fast actuators are still being designed, space for the actuators was reserved around stainless steel vacuum details during vacuum design. This was in order to not limit the actuator bandwidth due to chamber wall eddy currents, which persist significantly longer in a low-resistivity Cu chamber. The final actuator bandwidth is expected to be limited primarily by the power supply regulation loop. With some exceptions due to engineering constraints there will be four fast actuators available per achromat and plane in the 3 GeV ring, all located around a stainless steel chamber detail with circular cross section, 1 mm wall thickness and 25 mm inner diameter. The feedback loop logic itself will be implemented entirely in the Libera^®^ Brilliance+ system. Global exchange of BPM data will be carried out *via* the Global Data eXchange (GDX) modules in each Libera^®^ Brilliance+ unit, using a single optical fibre chain. Orbit correction calculations will then run in the Brilliance+ units on the field-programmable gate array (FPGA) available in the GDX module. The calculations will use a weighted response matrix to prioritize stability in the ID straights. Owing to the response matrix size the horizontal and vertical planes will be treated independently of one another. Once actuator set points have been calculated they will then be transmitted *via* RS485 links from the Brilliance+ units directly to the local actuator power supplies.

Both loops will communicate with one another in a manner similar to that in use at SOLEIL (Hubert *et al.*, 2005 ▶) in order to prevent the feedback loops fighting one another. The ‘slow’ loop will include a correction for each iteration to reduce the fast actuator strengths, while the ‘fast’ loop will use the most recent target orbit of the ‘slow’ loop as its reference. Further details are available from Sjöström *et al.* (2011 ▶).

## 7. Conclusions   

The MAX IV 3 GeV storage ring will be the first of a new breed of storage-ring-based light sources which make use of a multibend achromat lattice to reach unprecedented brightness and coherence. Commissioning of MAX IV will thus provide an opportunity for the validation of concepts that are likely to be essential ingredients of future diffraction-limited storage rings. Moreover, several future development possibilities aiming at further performance improvements are already under consideration (Leemann & Eriksson, 2013 ▶, 2014 ▶).

## Figures and Tables

**Table 1:** Main parameters of the MAX IV 3 GeV storage ring

Parameter	Value	Unit
Energy, *E*	3.0	GeV
Circumference, *C*	528	m
Maximum circulating current, *I*	500	mA
Main radio frequency, *f*_RF_	99.931	MHz
Number of long straights (available for IDs)	20 (19)	
Betatron tunes, β_*x*,*y*_	42.200, 16.280	
Natural chromaticities, 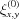	−49.984, −50.198	
Corrected chromaticities, 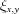	+1.0, +1.0	
Momentum compaction, α_c_, α_2_	3.06 × 10^−4^, 1.40 × 10^−4^	
Horizontal damping partition, *J*_*x*_	1.847	
Bare lattice emittance at zero current, ∊_0_	328	pm rad
Bare lattice natural energy spread at zero current, σ_δ_	0.769 × 10^−3^	
Bare lattice radiation losses	363.8	keV per turn

**Table 2:** Main parameters of the MAX IV 3 GeV ring RF system

Parameter	Commissioning	Final
Energy loss	360 keV	1000 keV
Current	200 mA	500 mA
Total SR power	72 kW	500 kW
Total RF voltage	1.0 MV	1.8 MV
Number of main cavities	4	6
Main cavity shunt impedance (*V* ^2^/2*P*)	1.6 MΩ	1.6 MΩ
Main cavity total copper losses	78 kW	169 kW
Main cavity coupling	1.9	4.0
Number of RF stations	4	6
Minimum RF station power	39 kW	114 kW
Total HC voltage	308 kV	478 kV
Number of HCs	3	3
HC shunt impedance	2.5 MΩ	2.5 MΩ
Total HC copper losses	6.3 kW	16 kW

## Footnotes

1 Note that this slightly increases the bare lattice emittance. Longitudinal gradients in bending magnets can be used to reduce the emittance (Nagaoka & Wrulich, 2007 ▶; Guo & Raubenheimer, 2002 ▶; Streun, 2004 ▶; Leemann & Streun, 2011 ▶), but this requires the bending radius to grow as the dispersion invariant  increases. For the soft-end bending magnets in MAX IV the zero-dispersion end of the bend is where the largest bending radii are.

2 An alternate optics has also been developed with linear chromaticity set to +4.0 as a fallback solution in case of instability issues during commissioning (Olsson & Leemann, 2013 ▶).

3 The emittance blow-up from IBS is calculated assuming the vertical emittance is adjusted to the 1 Å diffraction limit, *i.e.* 8 pm rad. If, however, a lower emittance coupling is chosen in order to improve ID brightness (Leemann & Eriksson, 2013 ▶), this blow-up becomes even more severe. For example, if skew quadrupoles are used to adjust the vertical emittance to 2 pm rad, the IBS blow-up for 500 mA becomes 69%, or 24% if bunch lengthening from Landau cavities is included.

4 On-axis injection requires injecting at a slight angle in the septum. The transfer line has been designed to include dipole correctors and diagnostics allowing adjustment of position and angle of the injected bunch in the septum.

5 The magnet system for the 3 GeV ring is described in detail in a companion paper in this issue (Johansson *et al.*, 2014 ▶). Early development and prototype work on these magnets are reported by Tarawneh *et al.* (2003 ▶, 2005 ▶). Here we briefly highlight the major aspects of the magnet design.

6 The vacuum system for the 3 GeV ring is described in detail in a companion paper in this issue (Al-Dmour *et al.*, 2014 ▶). Here we briefly highlight the major aspects of the vacuum design.
